# Two distinct classes of cochaperones compete for the EEVD motif in heat shock protein 70 to tune its chaperone activities

**DOI:** 10.1016/j.jbc.2022.101697

**Published:** 2022-02-09

**Authors:** Oleta T. Johnson, Cory M. Nadel, Emma C. Carroll, Taylor Arhar, Jason E. Gestwicki

**Affiliations:** 1 Institute for Neurodegenerative Disease, University of California San Francisco, San Francisco, California, USA; 2 Department of Chemistry, Beloit College, Beloit, Wisconsin, USA; 3 Department of Pharmaceutical Chemistry, University of California San Francisco, San Francisco, California, USA

**Keywords:** tetratricopeptide repeat, J-domain protein, molecular chaperone, protein–protein interaction, peptide–protein interaction, fluorescence polarization, ubiquitination, protein folding, CHIP, C terminus of Hsc70 interacting protein, CTD, C-terminal domain, DSF, differential scanning fluorimetry, FP, fluorescence polarization, G/F, glycine-phenylalanine–rich linker, Hsp72, heat shock protein 72 kDa, Hsc70, heat shock cognate 70 kDa, Hsp70, heat shock protein 70 kDa, Hsp90, heat shock protein 90 kDa, HOP, Hsc70 organizing protein, JD, J-domain, JDP, J-domain protein, NEF, nucleotide-exchange factor, NBD, nucleotide-binding domain, PPI, protein–protein interaction, PTM, posttranslational modification, SBD, substrate-binding domain, SAR, structure–activity relationship, TB, terrific broth, TBS, Tris-buffered saline, TEV, tobacco etch virus, TPR, tetratricopeptide repeat

## Abstract

Chaperones of the heat shock protein 70 (Hsp70) family engage in protein–protein interactions with many cochaperones. One “hotspot” for cochaperone binding is the EEVD motif, found at the extreme C terminus of cytoplasmic Hsp70s. This motif is known to bind tetratricopeptide repeat domain cochaperones, such as the E3 ubiquitin ligase CHIP. In addition, the EEVD motif also interacts with a structurally distinct domain that is present in class B J-domain proteins, such as DnaJB4. These observations suggest that CHIP and DnaJB4 might compete for binding to Hsp70’s EEVD motif; however, the molecular determinants of such competition are not clear. Using a collection of EEVD-derived peptides, including mutations and truncations, we explored which residues are critical for binding to both CHIP and DnaJB4. These results revealed that some features, such as the C-terminal carboxylate, are important for both interactions. However, CHIP and DnaJB4 also had unique preferences, especially at the isoleucine position immediately adjacent to the EEVD. Finally, we show that competition between these cochaperones is important *in vitro*, as DnaJB4 limits the ubiquitination activity of the Hsp70–CHIP complex, whereas CHIP suppresses the client refolding activity of the Hsp70–DnaJB4 complex. Together, these data suggest that the EEVD motif has evolved to support diverse protein–protein interactions, such that competition between cochaperones may help guide whether Hsp70-bound proteins are folded or degraded.

Members of the heat shock protein 70 (Hsp70) family of molecular chaperones play a critical role in maintaining protein homeostasis (aka proteostasis). These chaperones bind to misfolded or unfolded proteins and direct them to diverse processes such as protein folding, translocation, complex formation, and degradation (1, 2). Remarkably, this diversity of functions is enabled by a relatively simple structure: Hsp70s are composed of a nucleotide-binding domain (NBD), a substrate-binding domain (SBD), and an ⍺-helical lid domain (Fig. 1*A*) (3, 4). In eukaryotes, many cytosolic Hsp70s also bear a C-terminal unstructured region terminating in a conserved EEVD motif. These various domains are in allosteric communication; for example, ATPase activity in the NBD causes a conformational change that regulates binding of “client” proteins in the SBD (5, 6).

**Figure 1:**
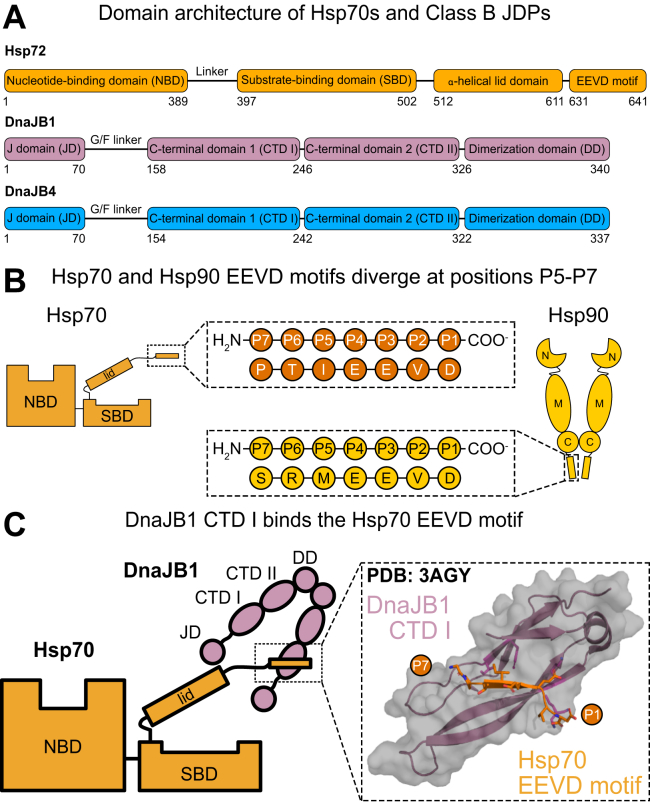
**Summary of the interactions between class B JDPs and the C-terminal EEVD motif of Hsp70.***A*, domain architecture of Hsp72 and the canonical, class B JDPs: DnaJB1 and DnaJB4. *B*, position nomenclature for the EEVD motifs of Hsp70 (Hsp72/HSPA1A) and Hsp90 (Hsp90α/HSP90AA1). In this nomenclature, the C-terminal aspartate is termed P1, such that the sequences of Hsp70s and Hsp90s begin to diverge in the P5 through P7 positions. *C*, cartoon and crystal structure representations of the interaction between Hsp70’s EEVD motif (*orange*) and DnaJB1’s CTD I (*purple*; PDB 3AGY (36)). Not shown in the cartoon schematic is the important interaction of the J-domain (JD) with Hsp70’s nucleotide-binding domain (NBD) and substrate-binding domain (SBD). Also, binding to only one CTD I in the dimer is shown for simplicity, but both are likely competent for this interaction.

Hsp70s rarely work alone. Rather, the diversity of Hsp70’s functions is imparted by cochaperones, such as J-domain proteins (JDPs) (7, 8), nucleotide exchange factors (NEFs) (9), and tetratricopeptide repeat (TPR) domain proteins (10). Some of these cochaperones, such as JDPs and NEFs, bind Hsp70s and stimulate cycles of nucleotide hydrolysis to regulate client binding (11, 12, 13, 14). In addition, cochaperones also act as adaptors, connecting Hsp70 and its clients to other cellular effector functions. For example, some NEFs and TPR proteins link Hsp70’s clients to protein degradation pathways (15, 16, 17). Thus, collaboration between Hsp70 and its cochaperones, mediated by a series of direct protein–protein interactions (PPIs), is critical for establishing the functional diversity of the chaperone complexes (18, 19). A key feature of this system, therefore, is that there are limited surfaces on Hsp70 for cochaperones to bind, such that the cochaperones must compete for shared sites to generate functionally distinct complexes. Accordingly, it is important to understand how Hsp70 binds its different cochaperones.

A major site of cochaperone binding is the EEVD motif that is present at the extreme C terminus of cytoplasmic Hsp70s (20, 21). Interesting, cytoplasmic Hsp90s, despite their dramatically different overall structure, also contain a conserved C-terminal EEVD motif (22). However, the Hsp70 and Hsp90 motifs differ at positions N-terminal to the EEVD sequence (Fig. 1*B*). Here, we will use the nomenclature in which the C-terminal aspartate is termed P1, whereas the P2 is the valine, etc. In this nomenclature, the cytoplasmic Hsp70s have an isoleucine at P5 (IEEVD), whereas the cytoplasmic Hsp90s have a methionine (MEEVD).

The binding of IEEVD and MEEVD motifs to TPR cochaperones have been extensively characterized (23, 24, 25, 26). Based on structural and biochemical evidence, a “carboxylate clamp” in the TPR domain makes electrostatic interactions to coordinate both the side chain of the P1 aspartic acid and the carboxy terminus (27, 28). A subset of these studies has specifically shown that some cochaperones have strong preference for Hsp70’s IEEVD or Hsp90’s MEEVD (29, 30, 31, 32, 33, 34). Furthermore, systematic mutations in Hsp70’s IEEVD motif have been used to reveal the structure–activity relationships (SARs) for binding to TPR proteins, such as Hsc70 organizing protein (HOP) and C terminus of Hsc70 interacting protein (CHIP) (33, 35).

More recently, it has become clear that some class B JDPs also bind to Hsp70’s EEVD motif (36, 37, 38). JDPs are categorized in three major classes (class A, B, and C) and are named for their conserved J-domain (JD) (39, 40, 41, 42), which binds Hsp70s near the interdomain linker between the NBD and SBD (43). This interaction requires an invariant HPD sequence within the JD and is responsible for the stimulation of Hsp70’s ATPase activity (7, 8, 11, 44). In addition to the JD, class B JDPs are typified by a glycine-phenylalanine–rich linker (G/F), two beta-barrel domains termed C-terminal domains 1 and 2 (CTD I/CTD II), and a dimerization domain (Fig. 1*A*) (8). CTD I and CTD II traditionally interact with prospective Hsp70 clients (37, 45, 46, 47), serving to recognize and deliver them to the chaperone. CTD I is also the site of interaction between class B JDPs and the EEVD motif (45, 48). This interaction was first characterized in the yeast JDP, Sis1, as well as the human JDP, Hdj1/DnaJB1 (Fig. 1*C*, PDB 3AGY) (36, 37, 38, 49, 50). Although binding of CTD I to the EEVD motif is typically weak (K_d_ ≅ 10–20 μM) (37, 51), it is functionally important. When the EEVD interaction is impaired, either by single point mutations in CTD I or truncations of Hsp70, the ability of the Hsp70 system to fold clients is inhibited (20, 37, 38, 52, 53). Recently, the EEVD interaction has also been demonstrated to relieve autoinhibition of the JD by the G/F linker, such that binding to the EEVD promotes ATPase activity, client refolding, and disaggregation of amyloids by Hsp70 (38, 54, 55).

Compared with the binding of TPR domains to the EEVD motif, less is known about the molecular determinants of the JDP–EEVD interaction. Structural studies have identified residues that contribute to functional activation of Hsp70s by class B JDPs (36, 37, 38, 49, 52, 53), but a detailed SAR for the EEVD–JDP interaction has not yet been described. Here, we characterize the determinants of the JDP–EEVD interaction using the representative class B JDP, DnaJB4. Using fluorescence polarization (FP) and differential scanning fluorimetry (DSF), we found that DnaJB4 binds selectively to the Hsp70 IEEVD motif but not Hsp90’s MEEVD sequence. Using truncations and mutations, we also found that DnaJB4 recognizes the carboxy terminus and has strong preferences for the P5 residue. Based on this knowledge, we developed an inactivating point mutation in Hsp70’s EEVD and used it to probe the functional importance of the interaction, showing that this secondary contact with DnaJB4 is critical for both ATPase and client refolding activities. We also confirmed that DnaJB4 can interfere with the function of the Hsp70–CHIP complex in ubiquitination assays and that, conversely, CHIP can partially disrupt the chaperone functions of the Hsp70–DnaJB4 complex. Together, these studies suggest that competition between distinct classes of cochaperones can tune the function of Hsp70 complexes.

## Results

### DnaJB4 binds Hsp70’s C-terminal EEVD motif

**Figure 2:**
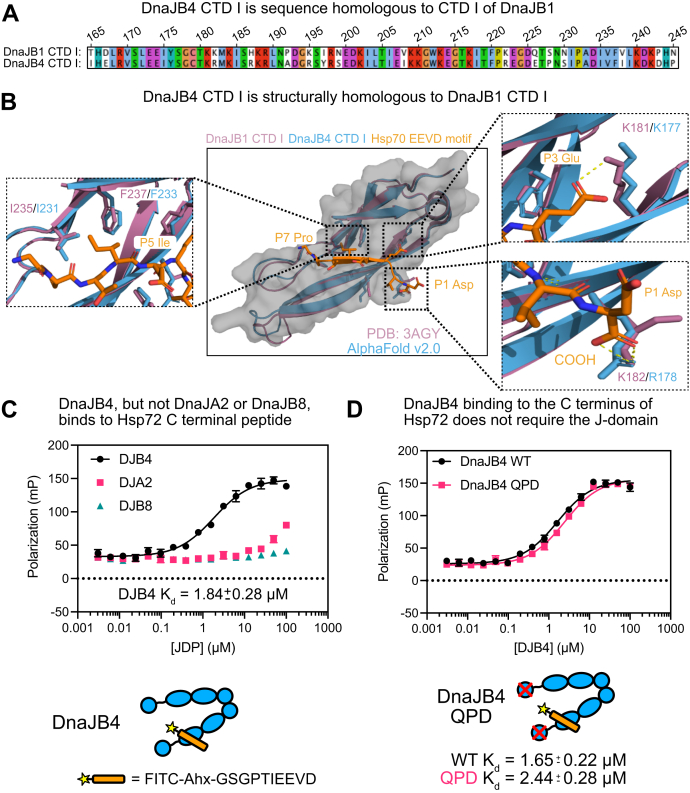
**DnaJB4 binds Hsp70’s C-terminal EEVD motif.***A*, sequence alignment of the CTD I domains of DnaJB1 and DnaJB4. *B*, structural alignment of the CTD I domains of DnaJB1 and DnaJB4 in complex with the Hsp70 EEVD motif. Critical residues for binding to Hsp70’s EEVD motif are shown as expanded images. Crystal structure of DnaJB1 CTD I in complex with Hsp70 EEVD motif (PDB 3AGY (36), *purple*, DnaJB1; *orange*, Hsp70 EEVD) was aligned to the predicted structure of DnaJB4 CTD I (AlphaFold v2.0 (56), *Blue*) in PyMol v2.x (Schrodinger). *C*, saturation binding of Hsp72 tracer to recombinant, human J-proteins, as measured by fluorescence polarization. The tracer sequence is included (*bottom*). The results are the average of four replicates, and error bars represent standard deviation (SD). Some error bars are smaller than the symbol. The K_d_ is shown as mean with a 95% confidence interval. *D*, saturation binding of Hsp72 tracer binding to WT DnaJB4 or QPD mutant. The results are the average of four replicates, and error bars represent SD. Some error bars are smaller than the symbol. K_d_ values are shown as the mean with a 95% confidence interval. CTD, C-terminal domain.

Before experimentally studying the molecular determinants of the EEVD–DnaJB4 complex, we first used the structure of an EEVD peptide bound to DnaJB1 (PDB 3AGY (36)), along with AlphaFold v2.0 (56), to create a model of the EEVD–DnaJB4 complex. Given the high sequence conservation between the CTD I domains (Fig. 2*A*), we hoped that this model could be used to make accurate predictions about which residues in the EEVD motif might be important for binding. Upon inspection, we noticed that a series of cationic residues coordinate the P3 glutamate side chain (DnaJB1 Lys181, DnaJB4 Lys177) and the EEVD carboxy terminus (DnaJB1 Lys182, DnaJB4 Arg178). We also observed hydrophobic contacts surrounding the side chain of the P5 isoleucine (DnaJB1 Ile235/Phe237 or DnaJB4 Ile231/Phe233). In contrast, other side chains in the EEVD, such as P1, P2, and P6, were solvent exposed and not as likely to make direct interactions. Thus, from the predicted structure, we were able to generate hypotheses about residues in the EEVD motif that make important contacts with CTD I of DnaJB4.

Then, to measure this interaction, we created a fluorescently labeled peptide derived from the last 10 residues of Hsp72/HSPA1A (*FITC-Ahx-GSGPTIEEVD*) and measured its interactions with recombinant, full length JDPs by FP. In this platform, DnaJB4 bound with a K_d_ of 1.84 ± 0.28 μM (Fig. 2*A*). We noted that this affinity is stronger than that estimated for the related protein, DnaJB1, by nuclear magnetic resonance (NMR) titrations (∼50 μM) (38). However, it is likely that the fluorophore and 6-carbon linker in the fluorescent peptide used here are responsible for the tighter apparent affinity (see below). To reveal any contribution of the J-domain to this interaction, we introduced the well-known QPD mutation (57) to DnaJB4 and compared binding to the fluorescent Hsp72 tracer. This mutation had no effect on binding, as both WT and the QPD DnaJB4 mutant bound with comparable affinities (WT K_d_ = 1.65 ± 0.22 μM; QPD K_d_ = 2.44 ± 0.28 μM) (Fig. 2*D*). To further test the selectivity of the interaction, we measured binding to DnaJA2, a member of the class A family of JDPs, and found that it had negligible affinity (Fig. 2*A*). Of interest, the fluorescent peptide also failed to bind DnaJB8, which is an atypical class B cochaperone that forms large oligomers (Fig. 2*A*).

### DnaJB4 binds Hsp70’s IEEVD motif but not the closely related MEEVD from Hsp90

**Figure 3:**
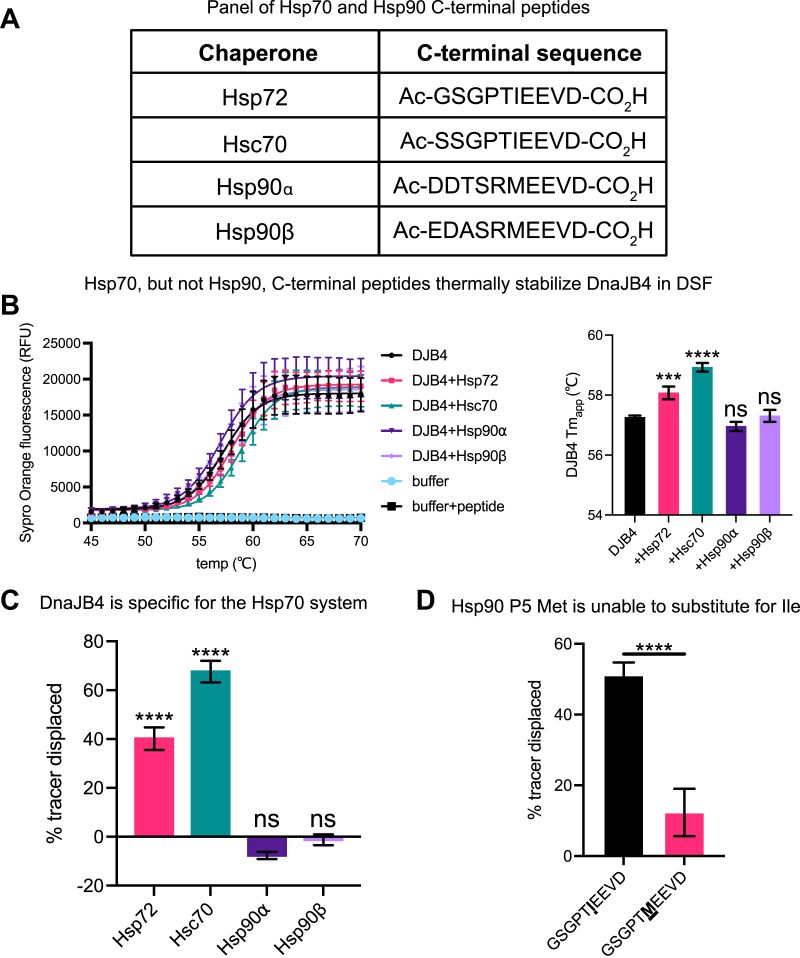
**DnaJB4 binds Hsp70’s IEEVD motif but not the closely related MEEVD from Hsp90.***A*, table listing the sequences of chaperone C-terminal peptides used in this study. *B*, differential scanning fluorimetry melting curves and apparent melting temperatures (T_m,app_) of 5 μM DnaJB4 in the presence of either a dimethyl sulfoxide (DMSO) control or various chaperone peptides (100 μM). Temperature-dependent unfolding was monitored by Sypro Orange fluorescence. The melting curves represent the mean Sypro Orange fluorescence ± SD (n = 4). Buffer alone and buffer + peptide samples were used as negative controls. The calculated DnaJB4 T_m,app_ values are mean ± SD (n = 4). Statistics were performed using unpaired Student’s *t* test (∗∗∗*p* < 0.001, ∗∗∗∗*p* < 0.0001, ns, not significant compared with DMSO control). *C*, FP experiment showing displacement of Hsp72 probe from DnaJB4 by various chaperone competitor peptides. Graph shows the mean tracer displacement relative to a DMSO control ± SD (n = 4). Statistics were performed using unpaired Student’s *t* test (∗∗∗∗*p* < 0.0001, ns, not significant compared with DMSO control). *D*, competition FP experiment comparing WT Hsp72 to a mutant in which the P5 Ile was replaced by Met. Graph shows the mean tracer displacement relative to DMSO control ± SD (n = 4). Statistics were performed using unpaired Student’s *t* test (∗∗∗∗*p* < 0.0001 compared with WT control).

Both cytosolic Hsp70s and Hsp90s terminate in EEVD motifs. To probe whether DnaJB4 could also bind Hsp90’s MEEVD motif, we generated unlabeled, *N*-acetylated 10-mer peptides corresponding to the C termini of the most abundant cytoplasmic chaperones from the Hsp70 (Hsc70/HSPA8, Hsp72/HSPA1A) and Hsp90 (Hsp90⍺ and Hsp90β) classes (Fig. 3*A*). Then, binding to DnaJB4 was measured using DSF. In the absence of peptide, DnaJB4 showed an apparent melting temperature (T_m,app_) of 57 ± 0.06 °C (Fig. 3*B*). Treatment with peptides from either Hsp72 or Hsc70 caused a positive shift of approximately 1 to 2 °C in the melt curves (Hsp72 T_m,app_ = 58 ± 0.21 °C; Hsc70 T_m,app_ = 59 ± 0.14 °C), suggesting that they bind to DnaJB4. Conversely, neither Hsp90⍺ nor Hsp90β peptides bound to DnaJB4 (Hsp90⍺ T_m,app_ = 57 ± 0.15 °C; Hsp90β T_m,app_ = 57 ± 0.20 °C). To confirm this result, we tested the same peptides as competitors in the FP assay, using the fluorescent Hsp72 peptide as the tracer. In this format, peptides from Hsp72 and Hsc70 again bound to DnaJB4 (% tracer displacement Hsp72 = 40.2 ± 4.6%, Hsc70 = 67.6 ± 4.4%), whereas Hsp90⍺ and Hsp90β peptides did not (Hsp90⍺ = −7.7 ± 1.5%, Hsp90β −1.3 ± 2.2%) (Fig. 3*C*). We did not observe complete liberation of the tracer by the unlabeled Hsp72 competitor, even at concentrations of 100 μM, supporting our hypothesis that the fluorophore and/or linker enhance tracer affinity relative to the unlabeled peptide. To specifically ask whether the P5 Ile/Met contributes to the dramatic difference between binding Hsp70- and Hsp90-derived peptides, we substituted the P5 Ile in the Hsp72 10-mer for Met and then measured binding to DnaJB4 by FP. Indeed, this single mutation was sufficient to weaken binding to DnaJB4 (Fig. 3*D*). Taken together, these data show a direct interaction between DnaJB4 and chaperone C termini that is selective for the Hsp70 system.

### Residue-level determinants of the JDP–EEVD interaction

**Figure 4:**
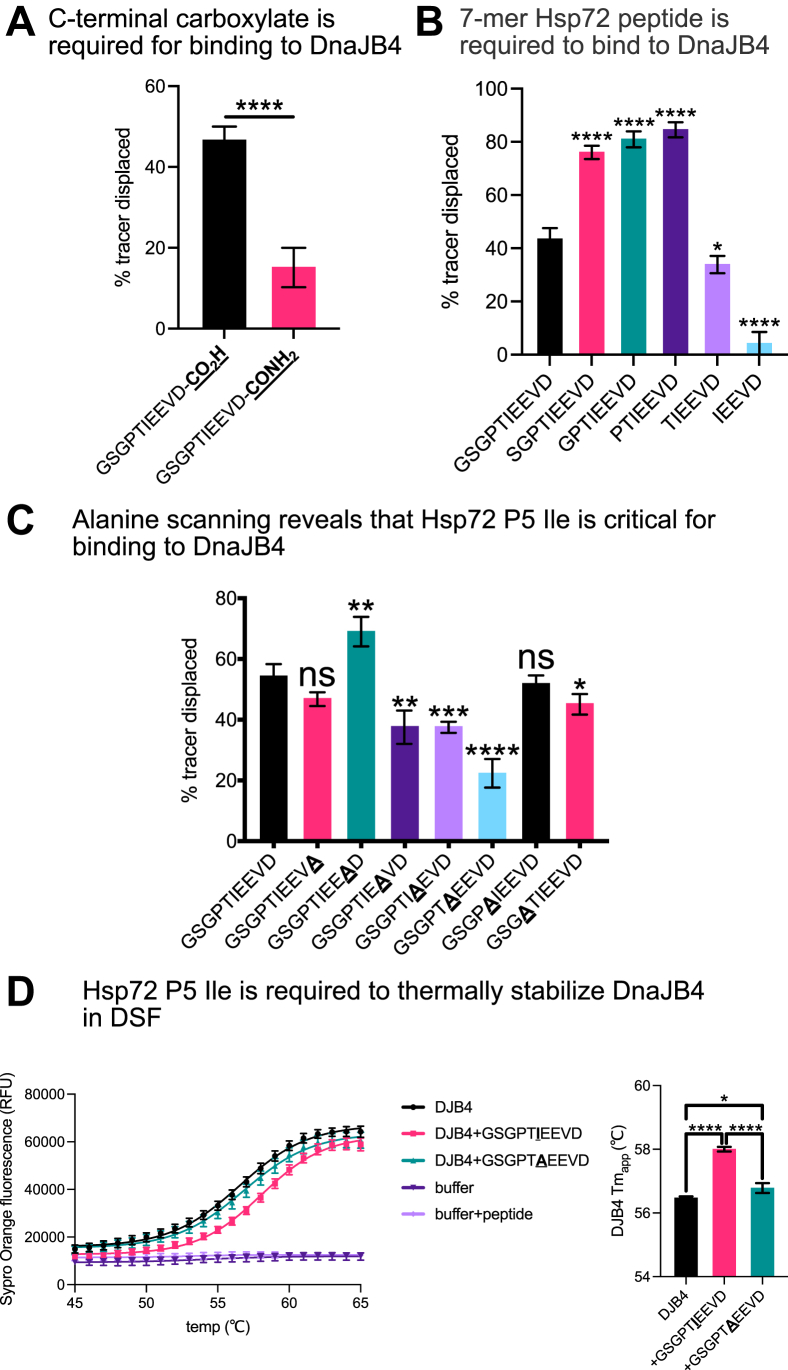
**Residue-level determinants of the JDP–EEVD interaction.***A*, competition fluorescence polarization (FP) experiment comparing displacement of Hsp72 tracer from DnaJB4 by WT or C-terminally amidated Hsp72 peptides. Graph shows mean tracer displacement relative to dimethyl sulfoxide (DMSO) control ±SD (n = 4). Statistics were performed using unpaired Student’s *t* test (∗∗∗∗*p* < 0.0001 compared with WT control). *B*, competition FP experiment comparing displacement of Hsp72 tracer from DnaJB4 by N-terminally truncated competitor peptides. Graph shows mean tracer displacement relative to DMSO control ±SD (n = 4). Statistics were performed using unpaired Student’s *t* test (∗*p* < 0.05, ∗∗∗∗*p* < 0.0001 compared with WT control). *C*, competition FP experiment comparing alanine scanning substitutions across the Hsp72 C-terminal sequence in binding to DnaJB4. Graph shows mean tracer displacement relative to DMSO control ±SD (n = 4). Statistics were performed using unpaired Student’s *t* test (∗*p* < 0.05, ∗∗*p* < 0.01, ∗∗∗*p* < 0.001, ∗∗∗∗*p* < 0.0001, ns, not significant compared with WT control). *D*, differential scanning fluorimetry experiment confirming the role of the P5 Ile in Hsp72 EEVD motif binding to DnaJB4. Temperature-dependent unfolding was monitored by Sypro *Orange* fluorescence at the denoted temperatures (°C). Melt curves are representative of the mean Sypro Orange fluorescence ±SD (n = 4). Buffer alone and buffer + peptide samples were used as negative controls. DnaJB4 T_m,app_ is represented as mean ± SD (n = 4). Statistics were performed using unpaired Student’s *t* test (∗*p* < 0.05, ∗∗∗∗*p* < 0.0001).

After establishing that DnaJB4 interacts with the Hsp70 C-terminus, we wanted to further delineate the molecular determinants of this PPI. The C-terminal carboxylate is obligate for EEVD binding to TPR domain cochaperones like CHIP (35); thus, we first asked whether the carboxylate might also be required for binding to DnaJB4. In FP competition assays, amidation of the carboxylate drastically reduced binding (Fig. 4*A*), suggesting that it is a critical feature. Next, we used truncations to probe how much of the 10-mer sequence was required. Removing the P10, P9, or P8 positions improved, rather than inhibited, binding to DnaJB4 (Fig. 4*B*). Truncation of the P7 Pro residue (TIEEVD), however, significantly weakened affinity, and truncation of the P6 Thr residue (IEEVD) further worsened it. These findings are consistent with our structural predictions (see Fig. 2*A*), wherein the last seven amino acids of Hsp70’s C terminus (PTIEEVD) are predicted to be necessary to span the entire PPI interface and the C-terminal carboxylate makes an important ionic interaction.

Having identified that the P1–P7 residues are required for the interaction with DnaJB4, we then performed an alanine scan of the 7-mer sequence to assess the individual contributions of each residue. The most dramatic effect was found at P5, where an alanine mutation significantly weakened binding (Fig. 4*C*). We confirmed this result using DSF, finding that the single P5 Ile to Ala mutation abrogated thermal stabilization of DnaJB4 (Fig. 4*D*). This result can be rationalized in the predicted structure, where the Hsp72 P5 isoleucine is “caged” by neighboring hydrophobic residues, Ile231 and Phe233, in DnaJB4 (see Fig. 2*A*). The only other positions that were sensitive to alanine mutation were the two glutamates at P3 and P4, with a more modest effect on the P7 proline. Together, these studies suggest that the carboxylate and the P5 Ile are most important for binding to DnaJB4 and that other side chains, especially P3, P4, and P7, make additional contributions to equilibrium binding affinity.

### DnaJB4 accommodates an expanded number of amino acids at P5 compared with CHIP

**Figure 5:**
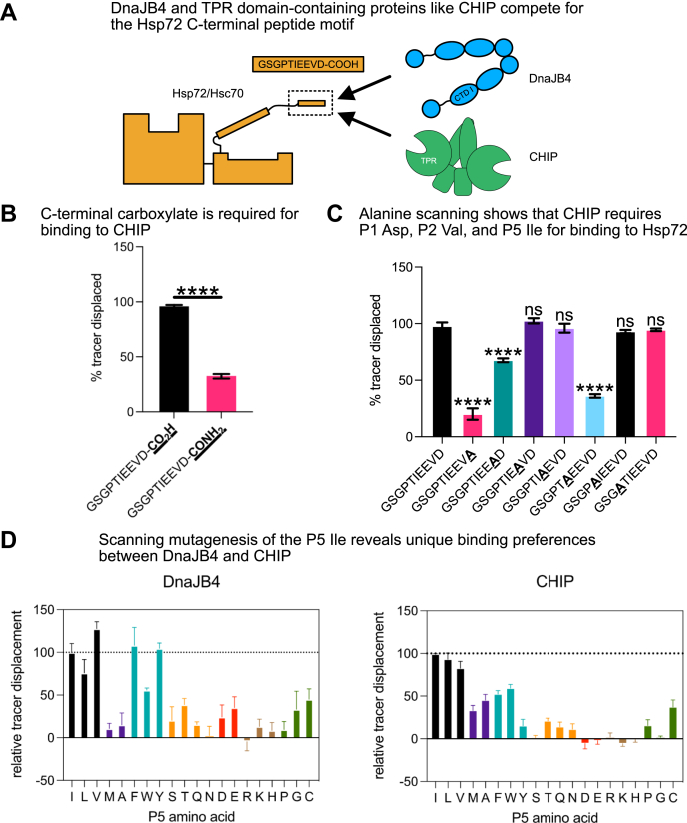
**DnaJB4 accommodates an expanded number of amino acids at P5, compared with CHIP.***A*, cartoon depicting competition for the Hsp70 C-terminal EEVD motif by DnaJB4 and CHIP. *B*, competition fluorescence polarization (FP) experiment comparing displacement of 20 nM Hsp72 tracer from 1.58 μM CHIP by 100 μM WT or C-terminally amidated Hsp72 peptides. Graph shows mean tracer displacement relative to dimethyl sulfoxide (DMSO) control ±SD (n = 4). Statistics were performed using unpaired Student’s *t* test (∗∗∗∗*p* < 0.0001 compared with WT control). *C*, competition FP experiment comparing alanine scanning substitutions across the Hsp72 C-terminal sequence in binding to CHIP. Graph shows mean tracer displacement relative to DMSO control ±SD (n = 4). Statistics were performed using unpaired Student’s *t* test (∗∗∗∗*p* < 0.0001, ns, not significant compared with WT control). *D*, competition FP experiment comparing all possible mutations at the P5 position of the Hsp72 EEVD motif in binding to DnaJB4 or CHIP. DnaJB4, 5 μM, or CHIP, 1.58 μM, was incubated with 20 nM WT Hsp72 tracer and 100 μM unlabeled competitor peptide. Tracer displacement was calculated relative to DMSO control and normalized to WT as 100% displacement. Graph shows mean relative tracer displacement ±SD (n = 4).

It is well known that TPR domain cochaperones recognize the P5 residue and the carboxy terminus in the EEVD motif (4, 24, 58). Because we found that the same positions are also critical for binding DnaJB4 (see Fig. 4), we expected that TPR cochaperones, such as CHIP, would compete for binding (as schematized in Fig. 5*A*). However, it was still not clear whether DnaJB4 and CHIP would have the same sequence requirements at P5. As an initial step in asking this question, we first wanted to repeat the reported binding studies with CHIP. This was an important step because the published experiments employed shorter, 5-mer peptides (35), and we wanted to facilitate direct comparisons with our findings in the DnaJB4 system using 10-mer peptides. Using the peptide with an amidated Hsp72 C terminus (GSGPTIEEVD-CONH_2_), we confirmed that the carboxylate was important for binding to CHIP in our FP assay (Fig. 5*B*). Then, we used the alanine scan peptides to show that the P1, P2, and P5 positions are indeed most important for binding CHIP (Fig. 5*C*). Thus, these results agree well with the reported findings. In analyzing these results, we did notice that the P3 and P4 glutamates, which are strictly conserved in the natural EEVD motifs, are dispensable for binding to CHIP. In contrast, these two positions are involved in binding to DnaJB4 (see Fig. 4*C*) and the P3 side chain is observed to make contacts with lysine 177 in DnaJB4’s CTD I (see Fig. 2*B*). Thus, evolutionary conservation of these glutamate residues might be primarily guided by the JDP interaction.

Next, to better understand the specific contributions at P5 for binding to both CHIP and DnaJB4, we tested Hsp72 peptides containing all natural amino acids at this position. With respect to CHIP binding, no substitution surpassed the native isoleucine in affinity. Furthermore, only branched-chain aliphatic residues (leucine and valine) were able to substitute for this residue, whereas charged and polar residues were strongly disfavored (Fig. 5*D*). With respect to DnaJB4, leucine and valine were able to substitute for isoleucine; furthermore, valine substitution modestly enhanced binding. Like CHIP, charged and polar residues were largely disfavored at the P5 position. Strikingly, though, certain aromatic residues (phenylalanine and tyrosine) could substitute for isoleucine. As previously mentioned, the P5 isoleucine is thought to project into a hydrophobic pocket created by residues Ile231 and Phe233 of DnaJB4 (see Fig. 2*A*). Thus, we hypothesize that phenylalanine or tyrosine may be accommodated and potentially stabilized by pi-stacking interactions with Phe233 in this pocket; however, further investigation is necessary to confirm this hypothesis. Collectively, these experiments suggest that DnaJB4 and CHIP use only partially overlapping molecular features to bind the EEVD motif.

### Mutations in the EEVD motif reduce collaboration between Hsp72 and DnaJB4

**Figure 6:**
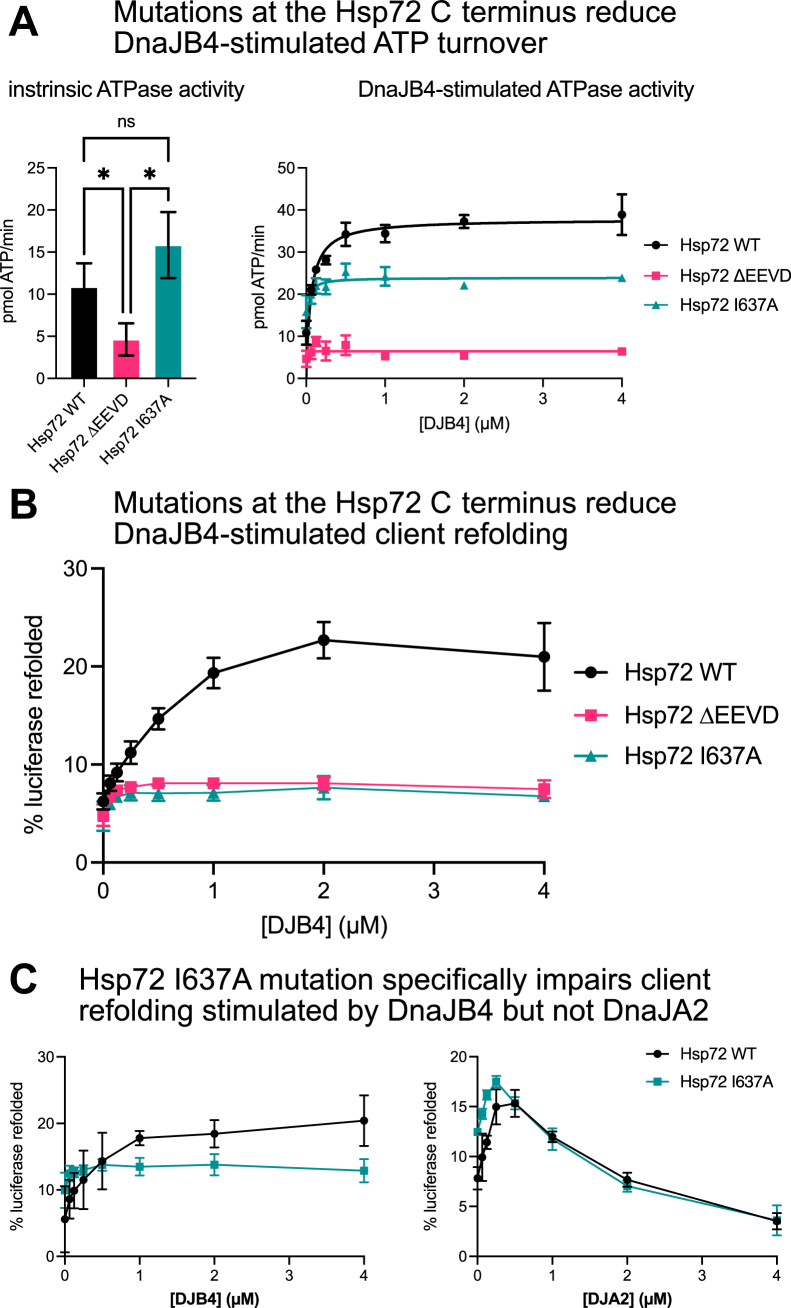
**Mutations in the EEVD motif reduce collaboration between Hsp72 and DnaJB4.***A*, ATP hydrolysis assay comparing the turnover rate of Hsp72 WT and mutants in the presence of DnaJB4, as measured by malachite green assay. The *left graph* shows mean intrinsic ATPase rate ±SD (n = 3) of the various Hsp72 mutants. The *right graph* shows mean ATPase rate ±SD (n = 3) of the various Hsp72 mutants in the presence of increasing concentrations of DnaJB4. Curves were fit according to Michaelis–Menten kinetics at steady state. Statistics were performed using unpaired Student’s *t* test (∗*p* < 0.05, ns, not significant). *B*, luciferase refolding assay comparing WT and mutant Hsp72 in the presence of DnaJB4. Refolding was measured by SteadyGlo luciferase reagent (see the Experimental procedures). The graph shows mean percent luciferase refolded relative to nondenatured luciferase control ±SD (n = 3). *C*, luciferase refolding assay comparing WT and I637A mutant Hsp72 in the presence of DnaJB4 or DnaJA2. The graph shows mean percent luciferase refolded relative to nondenatured luciferase control ±SD (n = 3).

Pioneering work by the Craig group showed that the interaction of Hsp70’s EEVD with class B JDPs is important for chaperone function (52, 53) and more recent structural studies have revealed that this effect is mediated by an allosteric release of autoinhibition that promotes JD function (38, 54). Here, we wanted to leverage our knowledge of DnaJB4’s SAR to probe these functional relationships in more detail. Toward this goal, we generated a mutant of full-length Hsp72 in which the EEVD motif was deleted (Hsp72 ΔEEVD). In addition, we created a point mutation of the critical P5 isoleucine residue (Hsp72 I637A), which significantly weakened, but did not abolish, binding to DnaJB4 (see Fig. 4). These two mutants were then tested in ATPase and luciferase refolding assays. First, we measured the intrinsic ATPase activities of the Hsp72 variants to create a baseline. As shown previously (20, 52), Hsp72 ΔEEVD had reduced intrinsic ATPase activity compared with the WT (WT = 10.9 ± 2.8 pmol ATP/min; ΔEEVD = 4.6 ± 1.9 pmol ATP/min). However, Hsp72 I637A had normal intrinsic activity (I637A = 15.8 ± 3.9 pmol ATP/min), so this mutant seemed better positioned for isolating the effects of DnaJB4 binding without the confounding effects on intrinsic turnover. Accordingly, we then measured the ability of DnaJB4 to stimulate ATPase activity by the Hsp72 variants. As expected, DnaJB4 stimulated the maximum ATPase activity (V_max,app_) of WT Hsp72 by ∼4-fold (37.8 ± 3.5 pmol ATP/min), at a half-maximal concentration (K_m,app_) of ∼0.06 μM. Conversely, DnaJB4 was unable to stimulate the Hsp72 ΔEEVD mutant (Fig. 6*A*), confirming previous reports that used other class B JDPs (52). Hsp72 I637A showed an intermediate level of activation, with a V_max,app_ of only ∼2-fold above baseline (23.9 ± 3.6 pmol ATP/min) and K_m,app_, ∼0.01 μM, showing that the affinity of the EEVD–CTD I interaction is important for ATP turnover.

Interesting, the effects of the I637A mutant were even more pronounced in luciferase refolding assays (Fig. 6*B*), in which both Hsp72 ΔEEVD and I637A were nearly completely impaired in the ability to coordinate with DnaJB4. Thus, the EEVD interaction is absolutely required to promote client refolding by DnaJB4, such that even the single alanine mutant could completely abrogate it. We speculate that this activity requires finely tuned kinetics. For example, DnaJB4 residence times may be shorter on the AEEVD motif compared with the IEEVD motif, leading to lower probability of proper coordination with Hsp72 during engagements with denatured luciferase (see Discussion). To verify that the point mutation did not affect the intrinsic ability to collaborate with JDPs in client refolding, we compared luciferase refolding by Hsp72 I637A in the presence of DnaJA2, which does not engage with the EEVD motif (38) (see Fig. 2*C*). As expected, Hsp72 I637A collaborated with DnaJA2 comparably with WT (Fig. 6*C*), suggesting that the EEVD interaction is only required for function with specific J-proteins, such as DnaJB4.

### Competition for the EEVD motif by cochaperones regulates chaperone functions

**Figure 7:**
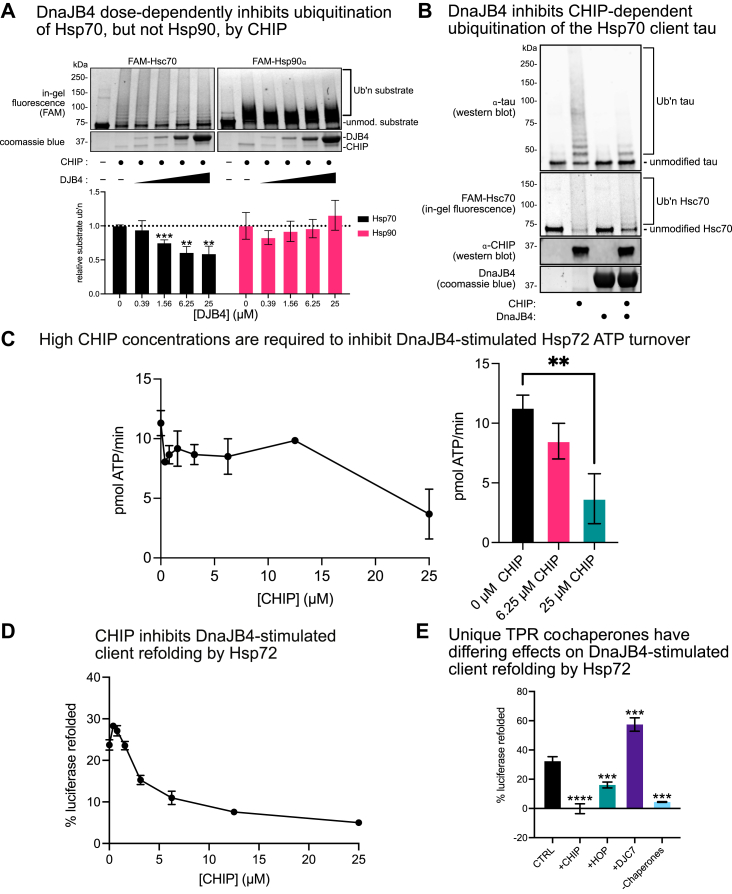
**Competition for the EEVD motif by cochaperones regulates chaperone functions.***A*, *in vitro* ubiquitination assay comparing ubiquitination of FAM-labeled Hsc70 or Hsp90⍺ by CHIP in the presence of increasing amounts of DnaJB4. Samples were separated by SDS-PAGE, and ubiquitination was analyzed by in-gel fluorescence, whereas CHIP and DnaJB4 were identified by staining with Coomassie blue. The graph shows mean substrate ubiquitination relative to no DnaJB4 control ±SD (n = 3). Statistics were performed using unpaired Student’s *t* test (∗∗*p* < 0.01, ∗∗∗*p* < 0.001 compared with no DnaJB4 control). *B*, *in vitro* ubiquitination assay comparing ubiquitination of MAPT/tau by CHIP in the presence of DnaJB4. Hsc70 was identified by in-gel fluorescence, whereas DnaJB4 was identified by staining with Coomassie blue. CHIP and tau were identified by Western blot. *C*, malachite green ATP hydrolysis assay comparing ATP turnover rate of WT Hsp72 in the presence of constant DnaJB4 and increasing concentrations of CHIP. Graph shows mean ATP hydrolysis rate ±SD (n = 3). Statistical analysis in the right panel was performed using unpaired Student’s *t* test (∗∗*p* < 0.01). *D*, luciferase refolding assay comparing ability of Hsp72 to refold client in the presence of DnaJB4 and CHIP. The graph shows mean percent luciferase refolded relative to nondenatured luciferase control ±SD (n = 3). *E*, luciferase refolding assay comparing the ability of Hsp72/DnaJB4 to refold client in the presence of various tetratricopeptide repeat (TPR) cochaperones. The graph shows mean percent luciferase refolded relative to non-denatured luciferase control ±SD (n = 3).

The complex of Hsp70s with CHIP is known to mediate the ubiquitination and degradation of client proteins (24, 59). Conversely, the complex of Hsp70s and DnaJB4 is most often associated with profolding functions (8). Thus, we hypothesized that competition between them might reciprocally inhibit these distinct functions. To test this idea, we first performed ubiquitination assays, wherein CHIP was used to ubiquitinate Hsp70 or Hsp90 *in vitro*. Consistent with previous findings (60, 61), both Hsp70 and Hsp90 were robustly ubiquitinated by CHIP, creating the expected laddering of high-molecular-weight, ubiquitinated species (Fig. 7*A*). Adding DnaJB4 to these mixtures resulted in dose-dependent inhibition of Hsp70 ubiquitination. As expected, DnaJB4 had no effect on Hsp90 ubiquitination, thus providing an important control. To test whether this competition might also affect the ubiquitination of Hsp70-bound clients, we similarly performed ubiquitination reactions using the well-documented Hsp70 client MAPT/tau (62) (Fig. 7*B*). Here, we observed robust ubiquitination of Hsc70 and tau by CHIP that was suppressed by the addition of DnaJB4, thus confirming our hypothesis that competition for the EEVD motif by DnaJB4 can suppress the prodegradation activity of CHIP.

Next, we explored whether CHIP might interrupt DnaJB4’s ability to stimulate Hsp72 ATPase activity. Indeed, we found that 40-fold excess of CHIP (25 μM) relative to DnaJB4 (625 nM) produced significant inhibition of DnaJB4-stimulated ATPase activity (Fig. 7*C*). This finding matches with previous observations, in which excess CHIP was required to block the activity of DnaJB1 in similar assays (63). Relatively high concentrations of CHIP may be required to suppress DnaJB4 function because multivalent contacts between Hsp70 and DnaJB4, mediated by both the JD and CTD I, effectively increase avidity.

We subsequently tested the ability of CHIP to suppress client refolding by the Hsp72–DnaJB4 complex. Indeed, titrations of CHIP into folding reactions showed that it is a potent inhibitor (Fig. 7*D*). The more pronounced ability of CHIP to suppress client refolding, compared with ATPase activity, is likely influenced by several factors, including CHIP’s described function as a “holdase” that can bind directly to unfolded clients, as well as CHIP’s preference for Hsp72 in the closed, ADP-bound state (64, 65). As binding to chaperone C termini is a shared feature of all TPR domain cochaperones (33), we reasoned that other TPR proteins may likewise compete with and alter the ability of DnaJB4 to promote luciferase refolding. Indeed, we found that HOP, another widely considered “profolding” cochaperone (66) suppressed luciferase refolding in the presence of DnaJB4 (Fig. 7*E*). This result was consistent with recent reports that HOP antagonizes luciferase refolding stimulated by DnaJB1 (67). Conversely, DnaJC7, a TPR cochaperone that also contains a J-domain, strongly promoted luciferase refolding by Hsp72, likely driven by its ability to stimulate ATP hydrolysis itself (68). Together, these studies confirm that TPR proteins and DnaJB4 compete for the EEVD motif to tune formation of Hsp70 complexes and influence chaperone function, but that the identity of the TPR co-chaperone is important.

### Pseudophosphorylation of the Hsp70 C terminus inhibits CHIP binding but has no effect on DnaJB4

**Figure 8:**
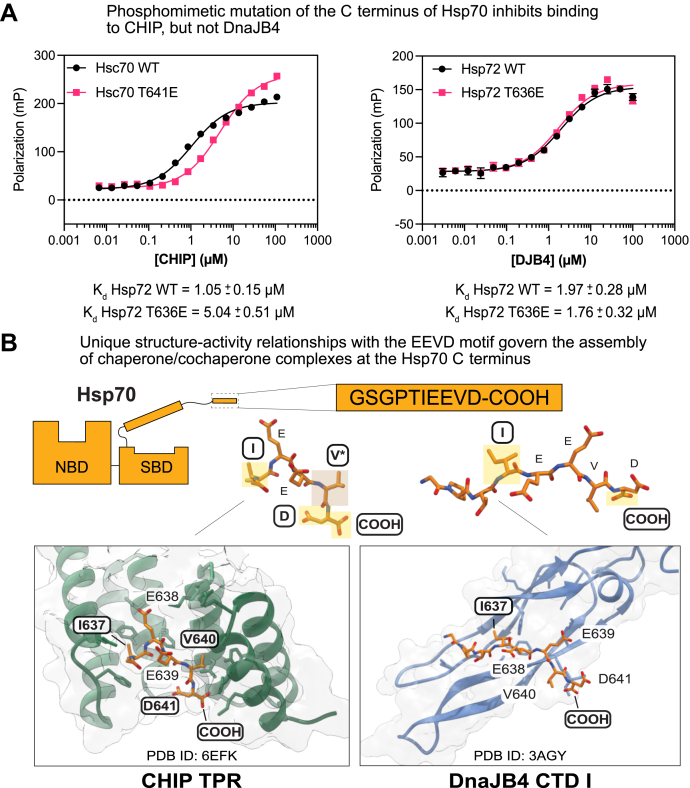
**Pseudophosphorylation of the Hsp70 C terminus inhibits CHIP binding but has no effect on DnaJB4.***A*, saturation binding fluorescence polarization experiments comparing WT or phosphomimetic T636E Hsp72 fluorescent tracer binding to CHIP (*left*) or DnaJB4 (*right*). Increasing concentrations of CHIP or DnaJB4 were incubated with Hsp72 fluorescent tracer for 30 min at room temperature. The results are the average of four replicates, and error bars represent SD. The K_d_ values for each condition are expressed as mean with a 95% confidence interval. *B*, comparison between the binding modes of Hsp70’s EEVD motif interacting with the CHIP or DnaJB4. The CHIP tetratricopeptide repeat (TPR) (*green*) orients the Hsp70 EEVD motif in a hooked or bent conformation, where it makes interactions with the P5 Ile, P2 Val, P1 Asp, and C-terminal carboxylate. Conversely, CTD I of DnaJB4 (*blue*) binds the Hsp70 EEVD in a linear orientation, with the P5 Ile and C-terminal carboxylate making important contacts. Residues important for binding to each cochaperone are highlighted on the crystal structures (CHIP TPR-Hsp70 EEVD: PDB 6EFK (35), DnaJB1 CTD I-Hsp70 EEVD: PDB 3AGY (36)).

Molecular chaperones are subject to myriad posttranslational modifications (PTMs), including AMPylation, methylation, acetylation, and phosphorylation (69, 70). Moreover, some PTMs have been directly linked to changes in the binding to cochaperones. Specifically, phosphorylation of the P6 threonine residue near the Hsp70 EEVD motif is known to inhibit binding of CHIP (29, 71). We confirmed this effect in our hands, as the mutation of P6 to the phosphomimetic, glutamic acid, resulted in a 5-fold weakening of the affinity of an Hsp72 peptide for CHIP (Fig. 8*A*). In contrast, the same peptide had no effect on binding to DnaJB4 (Fig. 8*A*), suggesting that phosphorylation might have a selective effect on CHIP but not DnaJB4. This observation is also supported by examination of the predicted binding modes for the EEVD motif when bound to CHIP or DnaJB4 (Fig. 8*B*, PDB 6EFK (35) and 3AGY (36)). When bound to CHIP, the EEVD motif is configured into an unstructured, bent conformation (26). In this binding mode, the P6 threonine is engaged in a hydrogen bonding interaction with the TPR domain, and phosphorylation of this residue is likely to generate electrostatic and/or steric clashes (35). Conversely, when bound to DnaJB4, the EEVD motif adopts a beta sheet conformation with the P6 threonine being relatively exposed to solvent (36). Phosphorylation of this residue is therefore unlikely to modulate binding to DnaJB4’s CTD I, consistent with the FP studies. Together, these results suggest that cells could use PTMs, especially phosphorylation of the C-terminus of Hsp70s, to tune binding at this PPI hotspot. More broadly, the drastically different configurations of the EEVD motif (*e.g.*, “bent” *versus* “linear”) when bound to these two domains further highlight the idea that molecular recognition by CHIP and DnaJB4 relies on only partially overlapping molecular features.

## Discussion

Interactions of Hsp70 with its cochaperones impart a strikingly diverse set of cellular functions to this molecular chaperone. Thus, a major goal in the proteostasis field is to understand when and where a particular complex between Hsp70 and its cochaperones will assemble. This is a challenging problem because there are approximately 13 NEFs (9), 44 JDPs (8), and 35 TPR cochaperone genes (27) and when these factors are combined with the six cytosolic Hsp70s (72), an upper limit of >120,000 unique possible combinations are possible. Although the true number of complexes is likely lower than this value because of restrictions in subcellular localization and tissue-specific expression, genetic and proteomic studies have supported the broad idea that cells contain many Hsp70 complexes (73, 74, 75). Thus, it is important to understand which cochaperones might compete and which molecular determinants are used to drive formation of these complexes.

Here, we focused on studying how the TPR domain proteins and class B JDPs converge on the Hsp70 EEVD motif (see Figs. 2*B* and 5*A*). This set of PPIs seemed especially important to understand because these cochaperones promote opposing functions of Hsp70, with JDPs directing the client to a profolding pathway and CHIP favoring client destruction (52, 63). Thus, competition for binding the EEVD could be central to the triage decisions made by the Hsp70 system. Indeed, we observed reciprocal inhibition of Hsp70 functions (see Fig. 7, *A* and *B*), suggesting that distinct classes of cochaperones regulate the functional outcomes of others *via* competition for the EEVD motif.

What controls the “decision” of Hsp70 to bind CHIP *versus* DnaJB4? It is easy to imagine that (at least) two parameters, relative affinity for the EEVD motif and relative abundance of a particular cochaperone, would combine to dictate which partner would bind at this PPI “hotspot.” Under the conditions tested, we found that CHIP has a slightly tighter affinity for the EEVD motif than DnaJB4 (Figs. 2*A* and 8*A*). Moreover, CHIP is abundant, is constitutively expressed, and has minimal tissue specificity (17, 76). These observations would suggest that CHIP is typically more available for binding to the EEVD motif, thereby potentially favoring client clearance over client folding. DnaJB4, however, is inducible under proteotoxic stress, greatly boosting its expression (77, 78). In addition, we observed a significant weakening of CHIP’s affinity when a phosphomimetic mutation is added to the EEVD motif, whereas DnaJB4 was unaffected (see Fig. 8*A*). Thus, signal transduction *via* transcription and/or phosphorylation would seem likely to favor DnaJB4 binding over CHIP. This could be why previous studies have observed that CHIP overexpression does not lead to client degradation, as might otherwise be predicted (79). Finally, we also observed tighter binding of DnaJB4 to the constitutive Hsc70 *versus* the stress-inducible Hsp72 (Fig. 3, *B* and *C*) and a dose-dependent protection of Hsc70 from CHIP-dependent ubiquitination by DnaJB4 (Fig. 7*A*). Thus, the relative levels of Hsc70 and Hsp72 might also dictate which complexes are formed and how quickly the chaperones are turned over. Finally, the relative kinetics of the EEVD interactions with TPR proteins and JDPs are not yet clear. Because Hsp70 functions require careful coordination of multiple, weak binding events (80), the relative association/dissociation rates and cochaperone residence times are likely to be important parameters, dictating both which complexes are formed and what allosteric signals are transmitted through those complexes. This may explain why ATPase stimulation of the Hsp72 I637A mutant is hampered; changes in binding kinetics due to the mutation may lead to a lower probability of allosteric communication to the distal Hsp72 NBD. Together, these findings suggest how cells might employ PTMs and transcriptional responses to fine-tune cochaperone affinities and concentrations, dictating which complexes are favored and, in turn, what Hsp70 functions are favored. In addition, this discussion must also acknowledge that there are other TPR and JDP cochaperones in cells (besides CHIP and DnaJB4), which provide additional layers of competition for the EEVD motifs.

Certain client proteins are also likely to tune these PPIs. For example, clients have been shown to bind CTD I and CTD II of JDPs (37, 45, 48), such that they would be expected to potentially compete with Hsp70’s EEVD motif. Accordingly, the production of unfolded clients by proteotoxic stress may directly impede EEVD binding to class B JDPs, perhaps promoting the formation of Hsp70–CHIP complexes. CHIP, on the other hand, directly interacts with a subset of substrates that are generated by caspase-dependent proteolysis (35). Briefly, caspase activity produces new C-termini that end in an aspartic acid, and some of these can resemble the EEVD motif. EEVD binding to CHIP requires a C-terminal aspartate (Fig. 5*C*); however, we found that EEVD binding to DnaJB4 does not require this side chain (Fig. 4*C*), suggesting that CHIP is more selective for neo–C-termini generated by caspase cleavage. Therefore, caspase activation may selectively displace CHIP, but not DnaJB4, from Hsp70. These scenarios highlight likely roles for clients in further shaping the distribution of proteostasis complexes in cells.

Chemical probes that can selectively perturb chaperone–cochaperone PPIs are desirable tools for dissecting the role of these complexes in cellular functions (81). Effective probes of this type would benefit from the ability to differentiate between closely related PPIs. Thus, we were interested in the finding that the EEVD motif binds to CHIP and DnaJB4 with partially distinct structural features. Specifically, the expanded side chain preferences of DnaJB4 for the P5 residue and its reliance on the P3 and P4 glutamates suggest that small molecules might preferentially block EEVD binding to this cochaperone over others. On the other hand, the requirement for a P1 aspartic acid and P2 valine in binding to CHIP, but not for DnaJB4, presents a potential opportunity for selectivity (see Fig. 4*C*). These predictions will require additional exploration, but it is compelling that the two classes of cochaperones “read” partially different chemical information in the EEVD motif.

## Experimental procedures

### Protein expression and purification

#### Class B J domain proteins

DnaJB4 and DnaJB8 were both expressed in *Escherichia coli* BL21 (DE3) Rosetta (New England BioLabs) cells from a pMCSG7 vector with N-terminal 6-His tag and tobacco etch virus (TEV) protease cleavable linker. Liter cultures of terrific broth (TB) were grown at 37 °C until the *A*_600_ reached 0.8. Cultures were then cooled to 18 °C, induced with 500 μM isopropyl beta-D-1-thiogalactopyranoside (IPTG), and grown overnight at 18 °C. Cell pellets were resuspended in His binding buffer (50 mM Tris pH 8.0, 10 mM imidazole, 750 mM NaCl) supplemented with cOmplete EDTA-free protease inhibitor cocktail (Sigma-Aldrich). Cells were lysed by sonication and pelleted by centrifugation, and the supernatant was applied to a 5-ml HisTrap Ni-NTA Crude column (Thermo Fisher Scientific). The column was washed with His binding buffer, followed by His wash buffer 1 (50 mM Tris pH 8.0, 30 mM imidazole, 750 mM NaCl, 3% EtOH) and His wash buffer 2 (50 mM Tris pH 8.0, 30 mM imidazole, 100 mM NaCl, 3% EtOH) supplemented with 1 mM ADP. The protein was eluted with a gradient elution from 0% to 100% His elution buffer (50 mM Tris pH 8.0, 300 mM imidazole, 300 mM NaCl). Eluent was supplemented with 1 mM DTT and TEV protease to remove the N-terminal His tag, and cleavage was allowed to proceed overnight at 4 °C and dialyzed to His Binding buffer. The protein was then buffer exchanged into His binding buffer and applied to Ni-NTA His-Bind Resin to remove His-tagged TEV protease. The protein was further purified by size exclusion chromatography using an AKTA Pure chromatography instrument (Cytiva) using Superdex 200 column (Cytiva) in Tris buffer (50 mM Tris pH 8.0, 300 mM NaCl).

#### Heat shock proteins 70

WT and mutant Hsp72/HSPA1A and Hsc70/HSPA8 were expressed in *E. coli* BL21(DE3) Rosetta cells from a pMCSG7 vector with N-terminal 6-His tag and TEV protease cleavable linker. Liter cultures of TB were grown at 37 °C until an *A*_600_ value of 0.6. Cultures were cooled to 20 °C and induced with 200 μM IPTG. Cultures were then grown overnight at 20 °C. Cell pellets were resuspended in binding buffer (50 mM Tris pH 8.0, 10 mM imidazole, 500 mM NaCl) supplemented with cOmplete EDTA-free protease inhibitor cocktail (Sigma-Aldrich). Cells were lysed by sonication and pelleted by centrifugation, and the supernatant was applied to HisPur Ni-NTA resin (Thermo Fisher Scientific). The resin was washed with binding buffer, washing buffer (50 mM Tris pH 8.0, 30 mM imidazole, 300 mM NaCl), and protein was eluted with elution buffer (50 mM Tris pH 8.0, 300 mM imidazole, 300 mM NaCl). Eluent was supplemented with 5 mM β-mercaptoethanol and TEV protease to remove the N-terminal His tag, and cleavage reaction was dialyzed into buffer A (25 mM Hepes pH 7.5, 5 mM MgCl_2_, 10 mM KCl) overnight at 4 °C. The protein was applied to a column packed with ATP-agarose (Sigma-Aldrich), and the column was washed with buffer A and buffer B (25 mM Hepes pH 7.5, 5 mM MgCl_2_, 1 M KCl). Protein was eluted with buffer A supplemented with 3 mM ATP.

#### C terminus of Hsc70 interacting protein

Recombinant human CHIP was expressed in BL21(DE3) (New England Biolabs) *E. coli* from a pMCSG7 vector with N-terminal 6-His tag and TEV protease cleavable linker and grown in TB to *A*_600_ = 0.6 at 37 °C. Cells were cooled to 18 °C, induced with 500 μM isopropyl β-D-1-thiogalactopyranoside (IPTG), and grown overnight. Cells were collected by centrifugation, resuspended in binding buffer (50 mM Tris pH 8.0, 10 mM imidazole, 500 mM NaCl) supplemented with protease inhibitors, and sonicated. The resulting lysate was clarified by centrifugation, and the supernatant was applied to Ni^2+^-NTA His-Bind Resin (Novagen). The resin was washed with binding buffer and His wash buffer (50 mM Tris pH 8.0, 30 mM imidazole, 300 mM NaCl) and then eluted from the resin in His elution buffer (50 mM Tris pH 8.0, 300 mM imidazole, 300 mM NaCl). Following, the N-terminal His tag was cleaved by overnight dialysis with TEV protease at 4 °C. The digested material was applied to His-Bind resin to remove cleaved His tag, undigested material, and TEV protease. The protein was further purified by size exclusion chromatography in CHIP storage buffer (50 mM Hepes pH 7.4, 10 mM NaCl), concentrated, flash frozen in liquid nitrogen, and stored at −80 °C.

DnaJA2 (82), 0N4R-Tau (82), Hsp90⍺ (29), HOP (29), and DnaJC7 (29) were purified as described.

### Peptides

Peptides were ordered from GenScript (95% purity by high-performance liquid chromatography). Fluorescence polarization tracer peptides were designed with a 5-carboxyfluorescein (5-FAM) moiety linked to the peptide N terminus *via* a six-carbon spacer (aminohexanoic acid). Unlabeled peptides were N-terminally acetylated to enhance stability and solubility. Unless specified, peptides bore an unmodified free carboxylate at the C terminus. Peptides were diluted in dimethyl sulfoxide (DMSO) to 10 mM stock solutions and stored at −20 °C.

### Fluorescence polarization

#### General

All FP experiments were performed in 384-well, black, low-volume, round-bottom plates at a final assay volume of 18 μl (Corning 4511). Polarization values in millipolarization units (mP) were measured at an excitation wavelength of 485 nm and an emission wavelength of 525 nm, with 100 flashes per read using a Spectramax M5 plate reader (Molecular Devices). All experiments were performed 2 times in quadruplicate. Experimental data were analyzed using GraphPad Prism 9. Saturation binding data were background subtracted, and curves were fit using the model [Agonist] *versus* response (three parameters). For competition experiments, data were background subtracted to tracer alone and normalized to DMSO control to determine relative tracer displacement.

#### Saturation binding to Hsp72 C-terminal probes

A sample of 20 nM Hsp72 tracers (FITC-Ahx-GSGPTIEEVD or FITC-Ahx-GSGPEIEEVD) was incubated with various concentrations of DnaJB4 (WT or QPD mutant) or CHIP in 2× dilutions (final [protein] = 110–0.013 μM) in binding buffer (JDP binding buffer = 50 mM Tris, 15 mM NaCl, 1 mM DTT, 0.01% Triton X-100, pH 8.0; CHIP binding buffer = 25 mM Hepes, 50 mM KCl, 0.01% Triton X-100, pH 7.4). The plate was covered from light and allowed to incubate at room temperature for 30 min prior to reading.

#### DnaJB4 FP competition experiments

Unlabeled peptides were assessed for the ability to compete with the Hsp72 tracer. Briefly, 100 μM peptides were incubated with 5 μM DnaJB4 and 20 nM Hsp72 tracer in JDP binding buffer (see above). The plate was covered from light and allowed to incubate at room temperature for 30 min prior to reading.

#### CHIP FP competition experiments

Mixtures of 1.58 μM CHIP and 20 nM Hsp72 tracer were incubated with 100 μM unlabeled competitor peptides in CHIP FP assay buffer (50 mM Hepes pH 7.4, 50 mM KCl, 0.01% Triton X-100). The plate was covered from light and allowed to incubate at room temperature for 30 min prior to reading.

### Differential scanning fluorimetry

DSF was performed with a 15-μl assay volume in 384-well Axygen quantitative PCR plates (Fisher Scientific) on a qTower (3) real-time PCR thermal cycler (Analytik Jena). Fluorescence intensity readings were taken over 70 cycles in “up-down” mode, where reactions were heated to desired temperature and then cooled to 25 °C before reading. The temperature was increased 1 °C per cycle. Each well contained 5 μM DnaJB4, 5 × Sypro Orange dye (Thermo Fisher), and 100 μM of peptide in JDP binding buffer. Fluorescence intensity data were truncated between 45 and 70 °C, plotted relative to temperature, and fit to a Boltzmann Sigmoid in Prism 9.0 (GraphPad). DnaJB4 apparent melting temp (T_m,app_) was calculated based on the following equation: Y = Bottom + ((Top-Bottom)/(1 + exp(*Tm*-*T*/Slope))).

### ATP hydrolysis assays

ATPase assays were carried out using the malachite green assay as described (14, 83). In brief, 1 μM Hsp72 and various concentrations of DnaJB4 were added to clear 96-well plates, and the reactions were initiated by addition of 2.5 mM ATP. Reactions were allowed to proceed for 1 h at 37 °C, after which they were developed using malachite green reagent and quenched with sodium citrate. Plate absorbance was measured at 620 nm, and a standard curve of sodium phosphate was used to convert the absorbance values to pmol ATP/μM Hsp72/min. V_max_ and K_m, app_ were derived as fit parameters to a modified Michaelis–Menten model (ATPase rate=V_max_∗[DnaJB4]/(K_m, app_+[DnaJB4])) where V_max_ reflects the maximal increased ATP hydrolysis conferred by DnaJB4 binding and K_m,app_ represents the half-maximal concentration of DnaJB4 binding to and stimulating the ATPase activity of Hsp72.

### Luciferase refolding assays

Experiments were performed as described (14). Briefly, *Renilla* luciferase (Promega) was denatured in 8 M GdnHCl for 2 h at room temperature. Hsp72 (1 μM) and denatured luciferase (100 nM) were diluted into a working concentration in buffer containing an ATP regenerating system (23 mM Hepes, 120 mM KAc, 1.2 mM MgAc, 15 mM DTT, 61 mM creatine phosphate, 35 units/ml creatine kinase, and 5 ng/μl bovine serum albumin, pH 7.4). A titration series of DnaJB4 was added, and the reaction was initiated with the addition of 2.5 mM ATP. For competition assays with DnaJB4, complexes of Hsp72 (1 μM), denatured luciferase (100 nM), and DnaJB4 (120 nM) were incubated with either a titration of CHIP or 10 μM of indicated TPR protein. In all cases, the assay was allowed to incubate for 1 h at 37 °C in white, 96-well flat-bottom plates (Corning 3990). Luminescence was measured using the SteadyGlo luminescence reagent (Promega), and percent refolded luciferase was calculated using a standard curve of 100 to 0 nM native luciferase.

### *In vitro* ubiquitination assays

Four 4 × stock solutions were prepared containing (i) Ube1 (R&D Systems) + UbcH5c/UBE2D3 (R&D Systems) (400 nM Ube1 and 4 μM UbcH5c), (ii) Ubiquitin (R&D Systems) (1 mM Ub), (iii) CHIP + substrate + DnaJB4 (4 μM CHIP, 4 μM substrate, varying concentrations of DnaJB4), and (iv) ATP + MgCl_2_ (10 mM ATP and 10 mM MgCl_2_) in ubiquitination assay buffer (50 mM Tris pH 8.0, 15 mM NaCl). CHIP + substrate + DnaJB4 solutions were allowed to equilibrate at room temperature for 30 min prior to initiating the reaction. Ubiquitination reactions were generated by adding 10 μl of each 4 × stock, in order from 1 to 4, for a final volume of 40 μl(100 nM Ube1, 1 μM UbcH5c, 250 μM ubiquitin, 2.5 mM ATP, 2.5 mM MgCl_2_, 1 μM CHIP and 1 μM substrate). Reactions were incubated at room temperature (10 min for chaperone ubiquitination, 30 min for tau ubiquitination), quenched in 20 μl 3 × SDS-PAGE loading buffer (188 mM Tris-HCl pH 6.8, 3% SDS, 30% glycerol, 0.01% bromophenol blue, 15% β-mercaptoethanol), and heated to 95 °C for 5 min. Samples were separated by SDS-PAGE on 4% to 20% polyacrylamide gels (Bio-Rad) and analyzed by in-gel fluorescence on a Chemidoc Imager (Bio-Rad) using the SYBR green fluorescence setting or by staining with Coomassie blue reagent. Quantitation of substrate ubiquitination was performed by densitometry analysis in ImageJ (NIH), which was thresholded to a no-CHIP control.

### Protein labeling with 6-carboxyfluorescein

Substrates for *in vitro* ubiquitination assays were labeled with 6-carboxyfluorescein (FAM) to enable in-gel fluorescence measurement of ubiquitination as described (35). Briefly, proteins were dialyzed into labeling buffer (25 mM Hepes pH 7.4, 50 mM KCl, 1 mM TCEP) and labeled by addition of 5 eq. of maleimide-FAM (Fisher Scientific 501143190) for 2 hours at room temperature. The reaction was quenched by addition of 1 mM DTT, and excess reagent was removed by iterative concentration and dilution over a 10-kDa MWCO microcentrifuge spin column (Pierce).

### Western blotting

Samples were separated by SDS-PAGE on 4% to 20% polyacrylamide gels and transferred to nitrocellulose blots using a Turbo Blot transfer system (Bio-Rad). Blots were blocked for 1 h at room temperature in Intercept Tris-buffered saline (TBS) Blocking Buffer (LI-COR), then incubated in primary antibody dissolved in TBS containing 0.05% Tween-20 (TBS-T) overnight at 4 °C. Blots were washed for 5 min 3× in TBS-T, then incubated in secondary antibody dissolved in Intercept T20 Antibody Diluent (LI-COR) for 1 h at room temperature. Blots were washed for 5 min 3× in TBS-T and imaged on an Odyssey FC Imaging System (LI-COR) at 700 and 800 nm wavelengths. Antibodies and dilutions used were as follows: ⍺-CHIP (1:2000, abcam #ab109103), ⍺-tau (1:1000, Santa Cruz Biotech #sc-1661060), IRDye 680RD goat anti-mouse secondary (1:10,000, LI-COR #926-68070), IRDye 800CW goat anti-rabbit secondary (1:10,000, LI-COR #926-32211).

## Data availability

All data are in the article. Source data are available upon request from the corresponding authors: Taylor Arhar, email: arhart@beloit.edu or Jason E. Gestwicki, email: jason.gestwicki@ucsf.edu.

## Conflict of interest

The authors declare that they have no conflicts of interest with the contents of this article.
